# Symmetry-mismatch reconstruction of genomes and associated proteins within icosahedral viruses using cryo-EM

**DOI:** 10.1007/s41048-016-0024-5

**Published:** 2016-05-27

**Authors:** Xiaowu Li, Hongrong Liu, Lingpeng Cheng

**Affiliations:** 1 College of Physics and Information Science, Synergetic Innovation Center for Quantum Effects and Applications, Hunan Normal University, Changsha, 410081 China; 2 School of Life Sciences, Tsinghua University, Beijing, 100084 China

**Keywords:** Viral genome, Cryo-EM reconstruction, Symmetry-mismatch

## Abstract

Although near-atomic resolutions have been routinely achieved for structural determination of many icosahedral viral capsids, structures of genomes and associated proteins within the capsids are still less characterized because the genome information is overlapped by the highly symmetric capsid information in the virus particle images. We recently developed a software package for symmetry-mismatch structural reconstruction and determined the structures of the genome and RNA polymerases within an icosahedral virus for the first time. Here, we describe the protocol used for this structural determination, which may facilitate structural biologists in investigating the structures of viral genome and associated proteins.

**Electronic supplementary material:**

The online version of this article (doi:10.1007/s41048-016-0024-5) contains supplementary material, which is available to authorized users.

## INTRODUCTION

Three-dimensional (3D) structural determination of viruses aids in elucidating viral molecular mechanism and pathogenesis. Cryo-electron microscopy (cryo-EM) and X-ray crystallography are two major methods used for the 3D structural determination of viruses. The major advantages of cryo-EM are that the specimens require no crystallization and are embedded in a thin vitrified ice of buffer thus preserving their near-native structure.

Recent instrumental and computational developments of cryo-EM have enabled the structural determination of viruses and other biological assemblies at near-atomic to atomic resolutions (Grigorieff and Harrison 2011; Bai et al. 2015; Bartesaghi et al. 2015), comparable to the structures determined using X-ray crystallography. A new generation of direct electron detection (DED) camera has significantly improved cryo-EM image quality. Compared with the traditional charge-coupled device (CCD) camera, DED camera allows reconstruction of higher resolution structures of biological complexes including viruses, while using fewer particle images (Veesler et al. 2013; McMullan et al. 2014; Bartesaghi et al. 2015). Icosahedral viral capsids were the first biological assemblies whose structures have been determined at near-atomic resolution using cryo-EM (Jiang et al. 2008; Liu et al. 2010; Cheng et al. 2011) due to their high (60-fold) symmetry and relatively large size, which promise a more accurate orientation determination and higher contrast of the particle images than other smaller low-symmetry biological assemblies.

Although the 3D structures of icosahedral viral capsids have been studied extensively by using both EM and X-ray crystallography for decades (Crowther 1971; Harrison et al. 1978; Baker et al. 1999; Thuman-Commike and Chiu 2000), precise structures of viral genomes and associated proteins within the capsids are still less characterized. The difficulty can be attributed to the fact that the viral genome and associated proteins are encapsidated in a high symmetric layered (or multi-layered) capsid. The high symmetry of the capsid is an advantage for determination of orientations of virus particle images using common-line based reconstruction algorithm (Crowther 1971; Fuller et al. 1996). However, when it comes to the structural determination of the genome and proteins within the capsid, the orientation determination of the non-symmetric genome information will be biased by the overlapping high-symmetry capsid information in the virus particle images. Here, we present our recently developed protocol and software package for the structural determination of icosahedral virus genome and associated proteins.

### Development of the layer-based cryo-EM image processing and symmetry-mismatch reconstruction method

Symmetry mismatches are present between viral capsids and genomes as well as within capsids. First attempts of symmetry-mismatch reconstruction made at virus structure determination were to reconstruct a unique tail at an icosahedral vertex of bacteriophages (Tao et al. 1998; Jiang et al. 2006). In the two previous studies, Jiang et al. and Tao et al. reconstructed the bacteriophage head structures taking advantage of the icosahedral symmetry, and then the icosahedral symmetry was further relaxed to lower symmetries to generate the reconstruction without symmetry imposition. Briggs et al. and Morais et al. reconstructed non-icosahedral structures at a Kelp fly virus vertex (Briggs et al. 2005) and a bacteriophage tail (Morais et al. 2001). They first boxed the vertex images from raw cryo-EM particle images with known icosahedral orientations, and then classified and reconstructed these boxed vertex images. However, their structural resolutions of non-symmetric regions of the viruses are relatively low, probably because the orientation determination of the target structural information was biased as mentioned above. In order to reduce the effect of the capsid information, Huiskonen et al. tried to remove the icosahedrally ordered capsid parts from the raw cryo-EM images of Cystovirus Φ8 by subtracting equivalent projections of the icosahedral model from the raw images (Huiskonen et al., 2007). The images of the RNA packaging motor boxed from the resulting image after subtraction were then classified and reconstructed to obtain a 15 Å resolution structure. The reconstruction resolution is low because the contrast transfer function (CTF) modulation of the images was not considered. In addition, the structures of encapsidated non-symmetric viral genomes and associated proteins remained unresolved.

In order to solve this problem, we developed a new symmetry-mismatch reconstruction method and for the first time we determined the structures of genome and polymerase within an icosahedral dsRNA cypovirus (Liu and Cheng 2015). In the subsequent sections, we have described a detailed protocol of this reconstruction method by using the cypovirus as an example.

### Applications and advantages of the protocol

The method has been released as a software package running under Microsoft Windows. The package is designed to reconstruct the 3D structure of lower or non-symmetry viral genome and associated proteins enclosed in higher symmetry viral capsid from 2D cryo-EM images. Nevertheless, it can be used for the reconstruction of other biological assemblies exhibiting similar symmetry mismatches. We believe that viruses have a functional state with a relatively organized genome structure that can be determined, and the key for the structure determination is how to catch this state biochemically.

### Limitations of the protocol

This protocol requires that the viral capsid is icosahedral and has been reconstructed at a high resolution from cryo-EM images of virus particles, of which the orientations and centers used for the capsid reconstruction have been determined by virus reconstruction software packages, for example, RELION (Scheres 2012), EMAN (Ludtke et al. 1999), and FREALIGN (Grigorieff 2007).

## EXPERIMENTAL DESIGN

Before reading the following protocol, we suggest the readers to refer to the basic principle of the protocol in the supplementary materials of our published paper (Liu and Cheng, 2015). The protocol includes two modules: the first module describes the steps to extract the non-symmetric genome structure information from the cryo-EM images by eliminating the symmetric capsid information. According to the approximation of weak phase objects, a cryo-EM image of a virus particle can be considered the sum of a genome image and a capsid image. Therefore, the genome image can be obtained by subtracting the capsid image, which can be obtained by projecting the 3D density map of the capsid on the capsid orientation and applying CTF modulation on the projection, from the cryo-EM image of a virus particle. The second module describes the steps to iteratively determine orientations of all genome images and reconstruction. Since the 60 equivalent orientations of the icosahedral capsid are known and the asymmetric genome structure has a fixed orientation related to the symmetric capsid, the correct orientation of the genome can be determined by searching the 60 equivalent orientations of the capsid (Liu and Cheng 2015). By using this protocol and software package, we could determine structure of genome and RNA polymerases within the cypoviruses.

## MATERIALS

Totally, 420 cryo-EM micrographs of a cypovirus were collected using Tecnai Arctica 200 kV electron microscope equipped with Falcon II camera at a magnification of 78,000 corresponding to a pixel size of 0.932 Å/pixel. Approximate 5000 virus particle images with a size of 1024 × 1024 pixels were boxed out from the 420 micrographs. The defocus value for each micrograph was determined using the CTFIT program in the EMAN package (Ludtke et al. 1999). In order to speed up the computational process, the images were subsampled by 2. All 2D image stacks and 3D density maps used in this protocol were stored in the MRC format.

## EQUIPMENT AND SOFTWARE SETUP

The following procedure requires a computer running Microsoft Windows and our symmetry-mismatch reconstruction software package. Unpacking the software package to a folder and setting a path to this folder in the Windows system are required before using the software package. This procedure assumes that users have experiences in cryo-EM image processing and single particle reconstruction.

## PROCEDURE

**Fig. 1:**
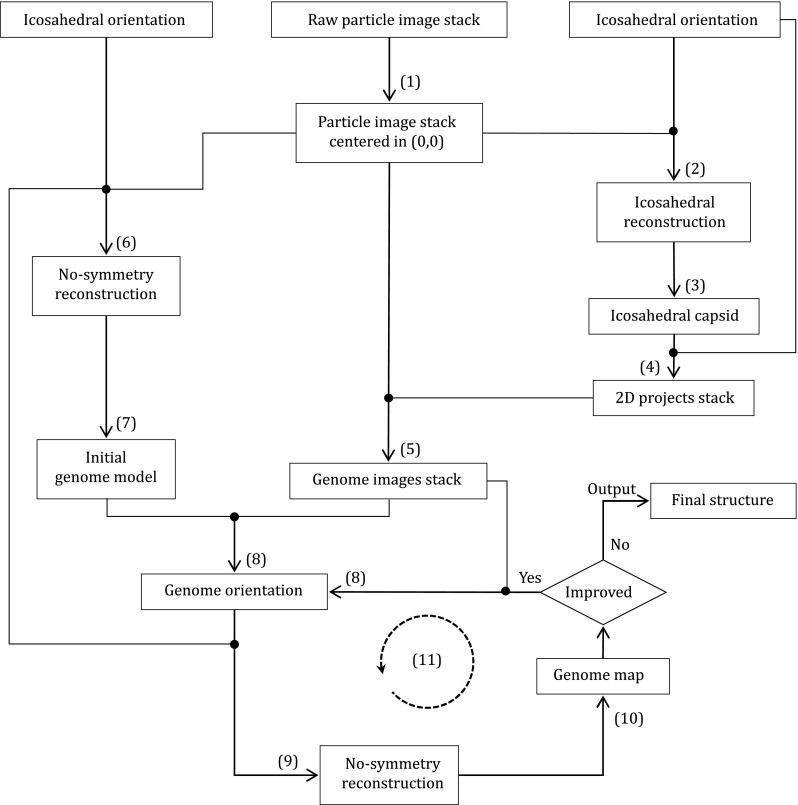
Flow chart of image processing and 3D reconstruction. (1)–(11) correspond to Steps (1)–(11) in the procedure

This section describes the step-by-step image processing procedure (Fig. 1). The lines in italic font are command lines, which must be input in the command prompt window in a directory where a stack of raw images are stored.

### Genome image extraction

(1) Moving the virus particle centers to the images centers. In order to avoid different definitions of the particle center in other reconstruction software packages, the centers of the virus particle images are moved to the center of the images.

*img2d* *rawImgBin2.stck* *outputimgstck*=*virusImgBin2.stck* *inputort*=*ort0.dat* *outputort*=*ort.dat norm*=*0,1 trans*=*y*

“rawImgBin2.stck” is a stack of the subsampled particle images with an image size of 512 × 512. “virusImgBin2.stck” is the output stack of the virus particle images whose particles are centered in the images. “ort0.dat” is a text file containing particle orientations, centers, and image defocus values (astigmatism) of these particle images (hereafter orientation file). “ort.dat” is a generated orientation file containing the same orientations and defocus values as those of “ort0.dat” but with the center parameters updated to 0,0. “norm=0,1” indicates that the images in “rawImgBin2.stck” are normalized to a mean value of 0 and a standard deviation of 1; “trans=y” is an option used to center the particles in the images.

The format of orientation file in this protocol is uniform. The first column contains the serial numbers of the virus particle images. The second, third, and fourth columns contain the Euler angles defining the orientations of these virus particles. The fifth and sixth columns contain centers defining the central positions of the virus particles in the images. The seventh column contains the correlation coefficients of the cross-correlation between the virus images and the model images. The eighth column contains serial numbers of the micrographs. The ninth, 10th, and 11th columns contain defocus values *X*, *Y*, and astigmatism angles of the particle images, respectively.

(2) Reconstructing a high-resolution density map of the capsid.

*recCartesian_fast virusImgBin2.stck ort.dat virusMap.mrc maxFR*=*220 imgmask*=*212 apix*=*1.864 applyCTF*=*y sym*=*I mincc*=*0.2 Cs*=*2.7 vol*=*200, ampw*=*0.1, norm*=*0,1*

**Fig. 2:**
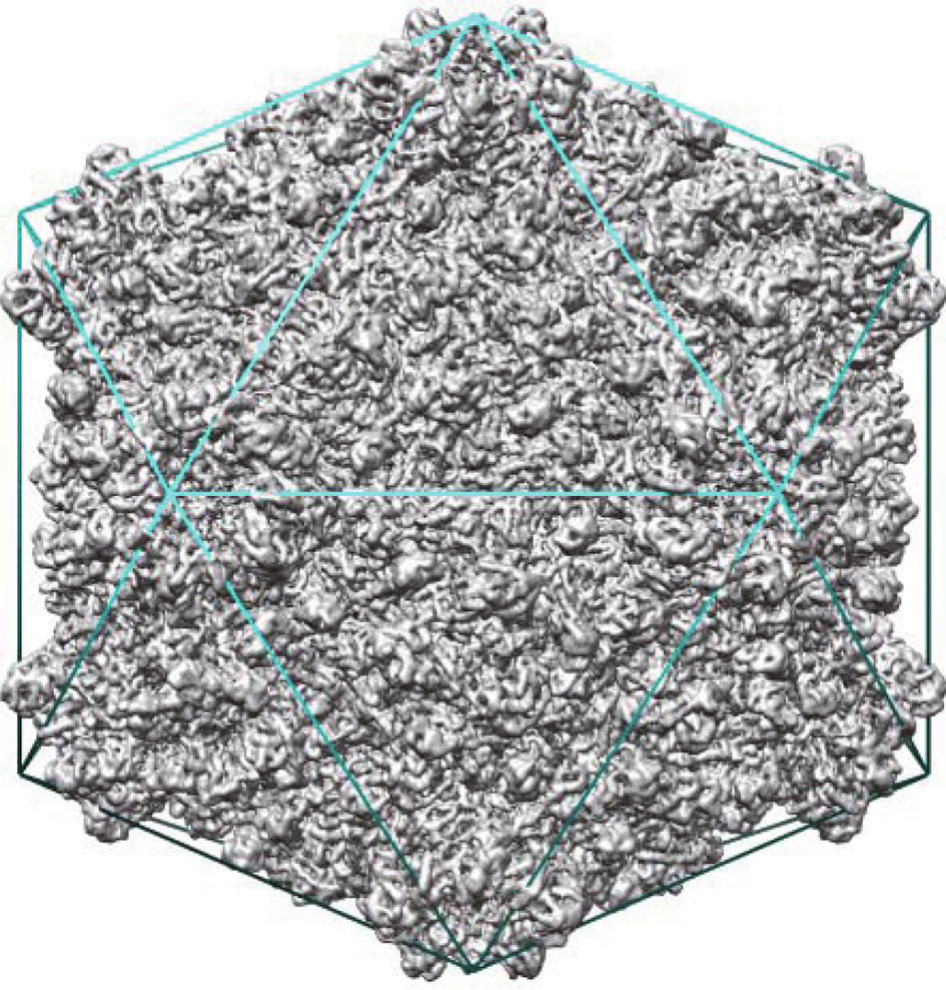
Structure of the cypovirus capsid

“virusImgBin2.stck” and “ort.dat” are stack of virus particle images and its orientation file generated in Step (1), respectively. “virusMap.mrc” is the output density map of virus; “maxFR=220”, “imagemask=212”, “apix=1.864”, “applyCTF=y” and “sym=I” specify the maximum Fourier radius (here, 220 corresponds to a resolution of 4.3 Å) used for reconstruction, mask radius for the virus particle images (in pixel), pixel size of the virus particle images, application of CTF correction, and applied symmetry during reconstruction (icosahedral), respectively. “Cs” and “vol” are the objective lens spherical aberration coefficient in mm and accelerating voltage of the electron microscope. “ampw=0.1” specifies the ratio of amplitude contrast. For cross-correlation (CC)-based particle orientation and center determination, “mincc=0.2” indicates that only those particle images with CC values higher than or equal to 0.2 are included in the reconstruction. For common-line-based particle orientation and center determination (Crowther 1971; Thuman-Commike and Chiu 2000), “mincc=0.2” indicates that only those particle images with cosine values of phase residuals higher than or equal to 0.2 are included in the reconstruction. The reconstructed density map of the cypovirus capsid is shown in Fig. 2.

(3) Masking the inner genome densities within the density map.

*img3d virusMap.mrc outputmap*=*capsidMap.mrc imask*=*136 mask*=*212*

“virusMap.mrc” is the density map of virus generated in Step (2). “imask” and “mask” specify radii of the genome and the capsid. This command eliminates the density within and outside the capsid and generates a density map of the capsid (“capsidMap.mrc”).

(4) Projecting the masked capsid density map according to the icosahedral orientation of each particle image.

*cent_slice_icos2f capsidMap.mrc ort.dat proj.stck mask*=*212 norm*=*0,1*

“capsidMap.mrc” is the capsid density map generated in Step (3). “proj.stck” is a stack of the projection images of the capsid map generated in this step. "ort.dat", which is generated in Step (1), indicates the projection orientations; therefore, these projection images have orientations identical to their corresponding virus particle images.

(5) Extracting genome images from the virus images.

*cc proj.stck stck2*=*virusImgBin2.stck ort2*=*ort.dat lp*=*220 norm*=*0,1 mask*=*212 applyCTF*=*y imgsubproj*=*genomeImg.stck trans*=*1,1*

“proj.stck” is the stack of capsid projection images generated in Step (4) and “virusImgBin2.stck” is the stack of virus images. “genomeImg.stck” is a stack of genome images generated in this step by subtracting the projection images from the virus particle images. Before subtracting, the program applies CTF to the projection images (“proj.stck”) and scales the pixel values on the projection images to the same grayscale with capsid information on the virus particle image. Pixel size, accelerating voltage, spherical aberration, ratio of amplitude contrast, B factor, and inner and outer radii values need to be input when the program is running. Amplitudes of 0.1 and B factor of 0 are recommended here. The two input radii define a circular region used for scaling the capsid information in virus particle images (“virusImgBin2.stck”) to the same grayscale with the corresponding capsid projections (“proj.stck”) (see Supplementary Fig. 13 of our previous paper (Liu and Cheng, 2015)). The inner and outer radii of 136 and 160 (in pixel) are recommended here. “trans=1,1” indicates that all genome will be moved to the center of the images. The extracted genome images (“genomeImg.stck”) will be used to determine the orientations of the genome images in Step (8).

### Initial model generation

(6) Reconstructing a complete virus structure (including the capsid and genome) using the virus particle images. The initial orientation for each virus particle image is randomly selected from the 60 icosahedral equivalent orientations of its capsid because the orientations computed from the virus capsid are all located within an asymmetric unit.

*icos2f_randort ort.dat ortRand.dat*

“ort.dat” contains orientations of virus particle images within an asymmetric unit. “ortRand.dat” contains orientations, each of which is randomly selected from the 60 icosahedral equivalent orientations of each orientation in the “ort.dat”.

*recCartesian_fast virusImgBin2.stck ortRand.dat capsidGenome.mrc maxFR*=*40 imgmask*=*212 norm*=*0,1 applyCTF*=*y Cs*=*2.7 vol*=*200 apix*=*1.864 ampw*=*0.1*

“virusImgBin2.stck” is the stack of virus images. “ortRand.dat” contains orientations of the virus images. “capsidGenome.mrc” is a generated density map of the complete virus structure.

(7) Generating a density map of the genome (initial model).

*img3d capsidGenome.mrc outputmap*=*genomeMap.mrc mask*=*136*

“mask=136” indicates that the densities out of radius 136 pixels will be masked. “genomeMap.mrc” is the generated density map of the genome.

### Orientation determination and 3D reconstruction of genome

(8) Projecting the 3D genome model to generate 60 projection images for each genome image according to the 60 equivalent icosahedral orientations of the capsid. The cross coefficients between each genome image and the 60 projection images are calculated here. The orientation of the projection image best-matched with each genome image is assigned the corresponding orientation to the genome image for further analysis.

*align_core genomeMap.mrc genomeImg.stck ort.dat genomeOrt.dat lp*=*15 mask*=*136 applyCTF*=*y norm*=*0,1 mode*=*cpc centshift*=*y apix*=*1.864 Cs*=*2.7 vol*=*200 ampw*=*0.1*

“genomeMap.mrc” is the structural model for the orientation determination of the genome images (“genomeImg.stck”). The orientations of the best-matched projection images are stored in “genomeOrt.dat”. “mode=cpc” indicates that the cross coefficient is calculated using cross phase correlation. “centshift=y” indicates that the center of the genome is allowed to shift when calculating cross coefficient. “lp=15” specifies the low-pass filter (in Fourier radius). “mask=136” indicates that only image region within a radius of 136 pixels in the genome images (“genomeImg.stck”) is used to calculate cross phase correlation.

This step is time intensive. Users can write a script to run it in parallel. For example, if the “genomeImg.stck” contains 5000 images, the script can be written as follows:

- *start align_core genomeMap.mrc genomeImg.stck ort.dat genomeOrt_1.dat lp*=*15 mask*=*136 applyCTF*=*y norm*=*0,1 mode*=*cpc centshift*=*y apix*=*1.864 Cs*=*2.7 vol*=*200 ampw*=*0.1 first* *1 last*=*1000*
- *start align_core genomeMap.mrc genomeImg.stck ort.dat genomeOrt_2.dat lp*=*15 mask*=*136 applyCTF*=*y norm*=*0,1 mode*=*cpc centshift*=*y apix*=*1.864 Cs*=*2.7 vol*=*200 ampw* *0.1 first*=*1001 last*=*2000*
- *start align_core genomeMap.mrc genomeImg.stck ort.dat genomeOrt_3.dat lp*=*15 mask*=*136 applyCTF*=*y norm*=*0,1 mode*=*cpc centshift*=*y apix*=*1.864 Cs*=*2.7 vol*=*200 ampw*=*0.1 first*=*2001 last*=*3000*
- *start align_core genomeMap.mrc genomeImg.stck ort.dat genomeOrt_4.dat lp* *15 mask*=*136 applyCTF*=*y norm*=*0,1 mode* *cpc centshift*=*y apix*=*1.864 Cs*=*2.7 vol*=*200 ampw*=*0.1 first*=*3001 last*=*4000*
- *start align_core genomeMap.mrc genomeImg.stck ort.dat genomeOrt_5.dat lp*=*15 mask*=*136 applyCTF*=*y norm*=*0,1 mode*=*cpc centshift*=*y apix*=*1.864 Cs*=*2.7 vol*=*200 ampw*=*0.1 first*=*4001 last*=*5000*

In each command, “first” and “last” indicate the first and last images to be processed in the “ort.dat”. Users can generate the combined “genomeOrt.dat” of all genome images by running *type genomeOrt_1.dat genomeOrt_2.dat genomeOrt_3.dat genomeOrt_4.dat genomeOrt_5.dat* > *genomeOrt.dat.*

(9) Reconstructing the genome using the newly assigned orientations and centers (“genomeOrt.dat”).

*recCartesian_fast virusImgBin2.stck genomeOrt.dat virusMap*-*1.mrc maxFR*=*40 imgmask*=*212 norm*=*0,1 applyCTF*=*y mincc*=*0.09 bound*=*3 Cs*=*2.7 vol*=*200 apix*=*1.864*

“bound=3” indicates that the genome images, whose centers shift more than 3 pixels, are not included in the reconstruction.

(10) Masking the capsid structure surrounding the genome structure.

*img3d virusMap*-*1.mrc outputmap*=*genomeMap*-*1.mrc mask*=*136*

The generated genome structure (“genomeMap.mrc”) serves as structural model for next round of orientation determination of the genome images.

**Fig. 3:**
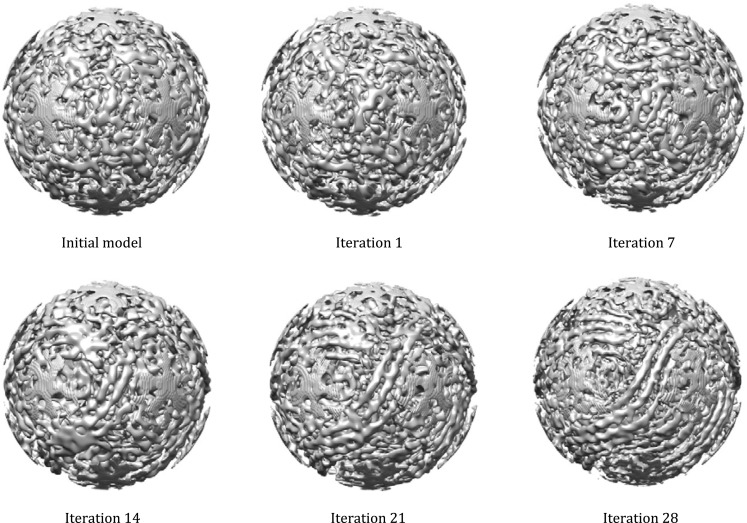
Density maps of the genome and RdRps from the initial model to reconstruction of 28 cycles. The dsRNA fragments are visible in the reconstruction of the 28th cycle

(11) Iterating Steps (8) to (10) until the orientations of each genome image stabilizes and no further improvement of the genome structure can be obtained. For each of the iteration, the output map generated in Step (10) is used as the structural model in Step (8). The “lp” of “align_core” and “maxFR” of “recCartesian_fast” can be improved steadily when the genome orientations in the “genomeOrt.dat” does not change during the iterations. The intermediate results during iterations are shown in Fig. 3.

## ANTICIPATED RESULTS

**Fig. 4:**
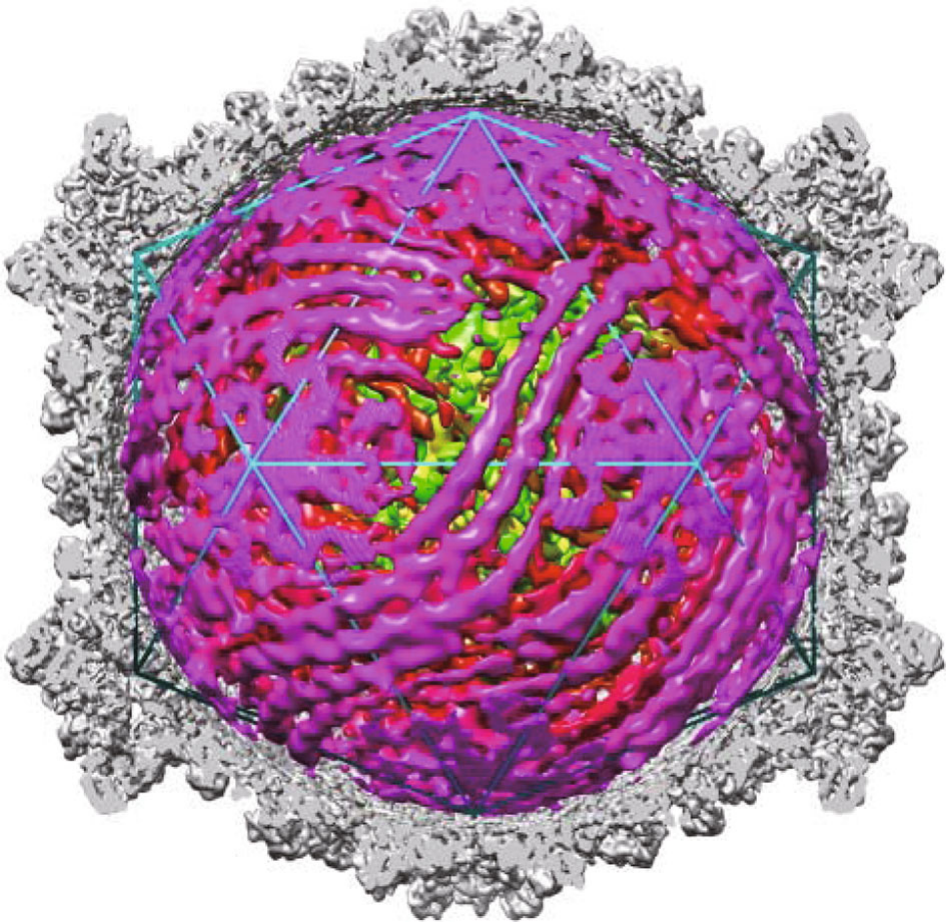
Structure of the genome within the capsid. Half of the icosahedral capsid (*gray*) is removed to show the structures of genomic dsRNA and RdRps (*purple*)

On performing this protocol, users can reconstruct a cryo-EM density map of the cypovirus genome structure (Fig. 4). The double helices of both dsRNA fragments located close to the inner capsid surface and interacting with the RNA polymerase can be observed. This protocol is also applicable for the genome structure determination of other icosahedral viruses with structurally homogenous genomes.

## Electronic supplementary material

Below is the link to the electronic supplementary material. The MRC image stack of virus particles can be downloaded from http://113.240.234.43:7080/.  

Supplementary material 1 (ZIP 4548 kb)
